# A Comparative Analysis of the Structure and Biological Properties of Films and Microfibrous Scaffolds Based on Silk Fibroin

**DOI:** 10.3390/pharmaceutics13101561

**Published:** 2021-09-26

**Authors:** Liubov Safonova, Maria Bobrova, Anton Efimov, Alexey Lyundup, Olga Agapova, Igor Agapov

**Affiliations:** 1 Academician V.I. Shumakov National Medical Research Center of Transplantology and Artificial Organs, Ministry of Health of the Russian Federation, Schukinskaya ul. 1, 123182 Moscow, Russia; saf.lyubov.msu@gmail.com (L.S.); mariabobrova.msu@gmail.com (M.B.); antefimov@gmail.com (A.E.); olya.agape@gmail.com (O.A.); 2 Educational Resource Center for Cellular Technologies, Peoples’ Friendship University of Russia, Miklukho-Maklaya str. 6, 117198 Moscow, Russia; information@rudn.ru

**Keywords:** silk fibroin films, biocompatible polymers, electrospinning, scanning probe nanotomography, full-thickness skin wound

## Abstract

A comparative analysis of the structure and biological properties of silk fibroin constructions was performed. Three groups of constructions were obtained: films obtained by casting an aqueous solution of silk fibroin and electrospun microfibrous scaffolds based on silk fibroin, with the addition of 30% gelatin per total protein weight. The internal structures of the films and single fibers of the microfibrous scaffolds consisted of densely packed globule structures; the surface area to volume ratios and volume porosities of the microfibrous scaffolds were calculated. All constructions were non-toxic for cells and provide high levels of adhesion and proliferation. The high regenerative potential of the constructions was demonstrated in a rat full-thickness skin wound healing model. The constructions accelerated healing by an average of 15 days and can be considered to be promising constructions for various tasks of tissue engineering and regenerative medicine.

## 1. Introduction

The development of biocompatible materials is one of the priorities of tissue engineering. Constructions fabricated with biomaterials interact with cells, which specify the purpose of the construction and ensure its physiological activity [1,2,3].

Scaffold material should correspond to the necessary rate of biodegradation and mechanical properties, imitate the native extracellular matrix structure as closely as possible and perform its functions without causing a toxic effect [4,5].

Currently, natural materials with biocompatibility and biodegradability without the formation of toxic degradation products are preferred [6,7,8]. Components of the native extracellular matrix, such as collagen, fibronectin, elastin, hyaluronic acid, etc., and polymers with a natural origin (alginates, chitosan, etc.) are widely used, as well as composites based on these materials [9,10]. One of these materials is gelatin, which is used as a composite additive to improve biocompatibility through its solubility and ability to utilize many novel methods, such as electrospinning [11].

However, utilization of the abovementioned materials has problems associated with the difficulties involved in structuring, insufficient mechanical strength and elasticity, low solubility, etc. In this regard, the study of the biological properties of silk fibroin from cocoons of the silkworm *Bombyx mori* and the development of scaffolds based on it are of particular interest [12]. Silk fibroin has a unique combination of properties and can be used in many tissue engineering areas, both alone and in composites [13,14].

The primary fibroin structure has the main repeating motif GAGAGS and accounts for 55% of amino acid residues. The remaining part has an amorphous structure and consists mainly of hydrophilic amino acid residues. The secondary structure presents antiparallel β-layers that are connected by hydrogen bonds. Amorphous regions form α-helices, which proportionally increase with protein hydration. The tertiary structure includes a heavy chain with a molecular weight of 390 kDa and a light chain of 26 kDa in a 1:1 ratio that is connected by disulfide bonds, as well as P25 glycoprotein of 30 kDa [15], which are assembled in a 6:6:1 ratio that corresponds to the form of the complex.

The main advantage of silk, as compared to other biocompatible materials, is its mechanical properties [16]. It ensures fibroin application as a frame-reinforcing component in various constructions [17,18] and as a composite additive in the polymers with insufficient mechanical strength [19,20,21].

A promising method for scaffold manufacturing is electrospinning, which is used to obtain porous fibrous mats with a fiber diameter from 50 nm to several micrometers. The fibrous structure significantly increases the cell proliferative activity and accelerates cell migration due to high porosity and a high surface area to volume ratio (SA:V). Electrospun scaffolds can be biomimetic in order to expand the scope of such constructions [22]. Many types of constructions that are based on silk fibroin are produced using the electrospinning method [23,24]. It allows for the fabrication of fibroin scaffolds with a constant orientation of fibers along one axis, for example, in neuron cultivation [25].

In the course of this research, the comparative analysis of silk fibroin-based films and microfibrous scaffold structures, biological properties and regenerative potential was performed, and novel data on the surface and internal structure was obtained.

## 2. Materials and Methods

### 2.1. Preparation of Silk Fibroin

Silk fibroin was obtained from *B. mori* silkworm cocoons [26,27], which were provided by the head of the State Scientific Institution of the Republican Scientific Research Station of Sericulture of the Russian Academy of Agricultural Sciences (Stavropol Krai, Zheleznovodsk, Russia) Bogoslovsky V.V. In the first stage, the cocoons were purified from sericin, 1 g of silk was boiled in a 500 mL of 2.52 M sodium bicarbonate aqueous solution in a water bath for 40 min and washed with 3.6 L of water. Then, the silk was boiled in 500 mL of water in a water bath for 30 min and washed with 3.6 L of distilled water. The last procedure was repeated 3 times. The purified silk fibroin was dried at room temperature.

### 2.2. Fabrication of Silk Fibroin Films

An aqueous solution of fibroin was prepared by boiling a solution of calcium chloride, ethanol and double-distilled water in a molar ratio of 1:2:8 and 130 mg/mL of silk fibroin for 5 h in a water bath [26,27]. Then, the solution was centrifuged for 7 min at 12,100× *g*. The supernatant was dialyzed against double-distilled water and centrifuged for 7 min at 12,100× *g*. The fibroin solution concentration was measured spectrophotometrically at a 280 nm wavelength. The films were obtained using the casting method [26]. To obtain a film with a diameter of 1 cm^2^, 100 μL of the silk fibroin solution with a concentration of 20 mg/mL was deposited on the surface of the polished Teflon and dried for 2 days at room temperature. The films were incubated in 96% ethanol for 15 min and were separated using a scalpel.

### 2.3. Fabrication of Silk Fibroin-Based Microfibrous Scaffolds

An aqueous solution of silk fibroin was dried in a Petri dish at room temperature. Dried silk fibroin and gelatin (Sigma-Aldrich, St. Louis, MO, USA) was dissolved in HFIP at a rate of 50 mg/mL. The solutions were centrifuged for 10 min at 12,100× *g*. To obtain composite scaffolds, the silk fibroin and gelatin solutions (supernatants after centrifugation) were mixed at the required ratio to the total protein concentration of 50 mg/mL.

Microfibrous scaffolds were obtained using the electrospinning method. The polymer solution was deposited on the surface of the fixed collector (steel plate) under an electric field with a voltage of 6.8–7 kV through a 22 G (inner diameter—0.4 mm) needle (for a silk fibroin solution), or through a 23 G (inner diameter—0.3 mm) needle (for a silk fibroin/gelatin solution). The solution feed rate was 0.1 mL/h and the needle collector distance was 7 cm. The scaffolds were dried at room temperature for 2 days and were then separated. For the cell adhesion and proliferation experiments, the deposition of the solution with similar parameters was performed on cover glasses attached to the collector.

### 2.4. The Analysis of the Surface Structure Using Scanning Electron Microscopy (SEM)

The samples of the constructions were fixed with a 2.5% of phosphate-buffered glutaraldehyde solution (pH 7.4) for 2 h at 4 °C in the dark; then, they were washed with phosphate-buffered saline. The samples were dehydrated using ethanol (10%, 20%, 50%, 70%, and 96% water solutions, 1 h in each concentration) and were incubated in acetone for 30 min.

The prepared samples were exposed to the critical point drying (31 °C, 72.8 kg/cm^2^) using the Quorum K850 instrument (Quorum Technologies, Lewes, UK). Sections of samples were prepared by scalpel and positioned inclined on the SEM platform for analysis. The dried samples were coated with a 10 nm gold layer in an argon atmosphere at an ion current of 20 mA and a pressure of 1 mbar using the Q150R ES rotary pumped coating system (Quorum Technologies, Lewes, UK). The samples were analyzed using the Tescan Vega3 SBU scanning electron microscope (Tescan, Brno, Czech Republic), with an operating voltage of 15 kV. Imaging was performed using VegaTC software (Tescan, Brno, Czech Republic).

### 2.5. The Analysis of the Film Surface Structure Using Atomic Force Microscopy

The samples were studied using a Solver P47-PRO atomic force microscope (NT-MDT Co., Zelenograd, Russia). Five samples of silk fibroin films were analyzed. Scanning was performed in a semi-contact mode with a scanning frequency of 0.3–0.4 Hz and an amplitude of 20–40 nm using NSC15/AL BS cantilever with a resonant frequency of 265–410 kHz and the force constant of 20–80 N/m (Mikromasch, Tallinn, Estonia). The scanned area was 11.324 × 11.324 μm^2^. The assembly of surface profiles and three-dimensional images and surface roughness estimations were performed using Nova 1.0.26.1433 software (NT-MDT Co., Zelenograd, Russia).

### 2.6. The Analysis of the Structure of Constructions by Scanning Probe Nanotomography (SPNT)

The samples were fixed with a 2.5% phosphate-buffered glutaraldehyde solution (pH 7.4) for 2 h at 4 °C in the dark and were washed with phosphate-buffered saline. Samples were dehydrated using ethanol (30%, 50%, 70%, 80%, and 96% water solutions, 10 min in each concentration). The samples were incubated in propylene oxide for 10 min twice and transferred into a 1:1 mixture of epoxy medium and propylene oxide, and then in a mixture ratio of 2:1. After that, the samples were incubated in an epoxy medium for 30 min at room temperature. Then, the embedding of the samples into the epoxy medium was performed and the samples were incubated at a temperature of 45 °C for 24 h, and then for another 48 h at a temperature of 60 °C.

A study of the samples’ structures was performed using the combined Ntegra Tomo system (NT-MDT Co., Zelenograd, Russia), which comprises a scanning probe microscope integrated with the ultramicrotome Leica EM UC6NT (Leica Microsystems GmbH, Vienna, Austria). Serial sections of the sample with 150 nm thicknesses were performed using Diatome UltraAFM 45 diamond knife (Diatome Ltd., Nidau, Switzerland) of the ultramicrotome with further measurement of the surface topography of each section by the atomic force microscope [28]. Measurements were performed in a semi-contact mode with a scanning frequency of 1 Hz. Silicon cantilevers NSG10 with a resonant frequency of 240 kHz (NT-MDT Co., Zelenograd, Russia) were used for measurements; the tip curvature radius was no more than 10 nm.

The images were obtained and processed, and the surface profiles were constructed using Nova 1.0.26.1433 software (NT-MDT Co., Zelenograd, Russia). To assemble three-dimensional reconstructions, the images were aligned at the scanning plane. Three-dimensional structures were analyzed by ImagePro AMS 6.0 software (MediaCybernetics Inc., Rockville, MD, USA), which includes the option of three-dimensional reconstruction.

### 2.7. Experiments with Cells

The 3T3 mice fibroblasts were used in the experiments. The cells were cultured in DMEM with a low concentration of glucose containing 10% fetal bovine serum, 0.324 mg/mL glutamine and 10 mg/mL gentamicin at 37 °C, 5% CO_2_. The cell monolayer was disaggregated using a Versen solution with the addition of 1.4 mg/mL trypsin; the cells were apportioned in a 1:3 ratio.

The analysis of the cytotoxicity of all the samples was performed using the MTT test [29]. The fibroblasts were cultured in a 96-well plate at 37 °C, 5% CO_2_ for 3 days to obtain the cell monolayer in the plates. Then, the culture medium was changed, and the samples of sterile constructions (0.3 × 0.3 cm^2^) were placed into the wells. The plates were incubated at 37 °C, 5% CO_2_. The MTT test was performed on the 3rd, 5th and 7th day of the experiment. A volume of 60 μL of 5 mg/mL MTT solution per well was added. The plates were incubated at 37 °C, 5% CO_2_ for 4 h. The samples were removed, and the plates were centrifuged for 5 min at 885× *g*. The formazan precipitate was dissolved in 300 μL of dimethyl sulfoxide and the optical density was measured at 540 nm.

To investigate cell adhesion and proliferative activity, the films were cut by hole puncher with 5 mm diameter of hole and were positioned in the wells of 96-well culture plates. Microfibrous scaffolds on cover glasses were positioned in Petri dishes with a diameter of 3.5 cm. The constructions were sterilized using 70% ethanol for 30 min and then irradiated with ultraviolet for 30 min. After that, the constructions were washed with sterile phosphate-buffered saline 3 times and incubated in the medium for 30 min.

The cell suspension in the medium was transferred to plates and Petri dishes at a rate of 1000 cells per well in 300 μL and 20,000 cell per Petri dish in 4 mL and incubated at 37 °C, 5% CO_2_.

Cell adhesion (1 day) and proliferative activity (5, 7 days) were evaluated by Carl Zeiss Axio Vert.A1 microscope (Zeiss, Jena, Germany). The samples were washed twice with a phosphate-buffered saline and stained with a DAPI fluorescent dye. An amount of 3 μg aqueous DAPI solution was added at a rate of 2 mL per Petri dish or 150 μL per well and incubated at 37 °C, 5% CO_2_ for 5 min. Then, the samples were washed twice with a phosphate-buffered saline. Cell images were obtained and processed using ZEN 2.3 (blue edition) software (Zeiss, Jena, Germany).

### 2.8. Full-Thickness Skin Wound Healing of a Wistar Rat

Male Wistar rats weighing 200–300 g were used in the experiment. The rats were isolated from each other in single cages with free access to water and food. All animal experiments were performed in accordance with European Convention for the Protection of Vertebrate Animals Used for Experimental and other Scientific Purposes (ETS); Directive 2010/63/EU and approved by the Local Ethical Committee of V.I. Shumakov Federal Research Center of Transplantology and Artificial Organs.

All operations with animals were performed under inhalation ether anesthesia, which was provided with desiccator at a rate of 50 mg/kg body weight. The animals were in spontaneous respiration with a frequency of 75 ± 10 respiratory cycles per minute, which corresponds to the surgical stage of anesthesia.

The animals were divided into 4 groups (Table 1). In group 4 (control group), all the same surgical procedures were performed with animals, and the wound was covered with a sterile gauze dressing.

Modeling of the full-thickness skin wound was performed as follows [30]. Hairs of rat back were removed, and the skin was treated with a 0.05% chlorhexidine solution. Next, the wound with a diameter of 15 ± 1 mm was made and treated with a 0.05% chlorhexidine solution. The depth of damage corresponded to the thickness of the rat skin, including epidermis, dermis and hypodermis.

Films were sterilized with 70% ethanol for 12 h; microfibrous scaffolds were sterilized for 1 h with an ultraviolet. In group 1, the film was placed on the wound surface in a wet form in order to prevent the formation of film microdefects and was not sutured due to its adhesion to the wound area. In groups 2 and 3, a microfibrous scaffold was placed in a dry form and was not sutured.

Then, the dressing wound was treated with a 0.05% chlorhexidine solution and coated with sterile gauze dressing, which was removed on the 3rd day of the experiment.

The widest diameter of the wound (d) was measured, and the wound healing area (A) was calculated on the 0th, 3rd, 7th, 14th, 21st, 23rd, 28th and 40th days of the experiment, according to the formula:

$$A=\frac{d_{\left(0\right)}{\;-\;d}_{\left(0,3,7,14,21,23,28,40\right)}}{d_{\left(0\right)}}\;\times \;100\%$$

A—wound healing area; $d_{\left(0\right)}$—initial wound diameter (day 0); $d_{\left(0,3,7,14,21,23,28,40\right)}$—wound diameter on the control day of the experiment.

Then, the curves of the dynamics of the wound closure process were plotted.

### 2.9. Histological Evaluation

Samples of rat skin that were 20 ± 3 mm in size were fixed using a mixture of formalin, ethanol and acetic acid in a volume ratio of 4:1:0.3 and were embedded into paraffin. Paraffin blocks were sliced; sections with thicknesses of 10 μm were obtained by rotary microtome Microm HM 325 (Thermo Scientific, Waltham, MA, USA), stained with hematoxylin-eosin, embedded in Canada balsam and analyzed by Carl Zeiss Axio Vert.A1 microscope (Zeiss, Jena, Germany). Images were obtained by Axiocam 305 color digital camera (Zeiss, Jena, Germany) and processed by ZEN 2.3 (blue edition) software (Zeiss, Jena, Germany).

### 2.10. Statistical Processing of Results

Data was processed using an analysis of variance (ANOVA). The statistical significance of the results was evaluated using the Mann–Whitney U test. The level of statistical significance α was equal to 0.05.

## 3. Results

In the course of this research, three groups of samples were fabricated by two different methods (Table 1).

### 3.1. Investigation of Construction Structure

**Figure 1:**
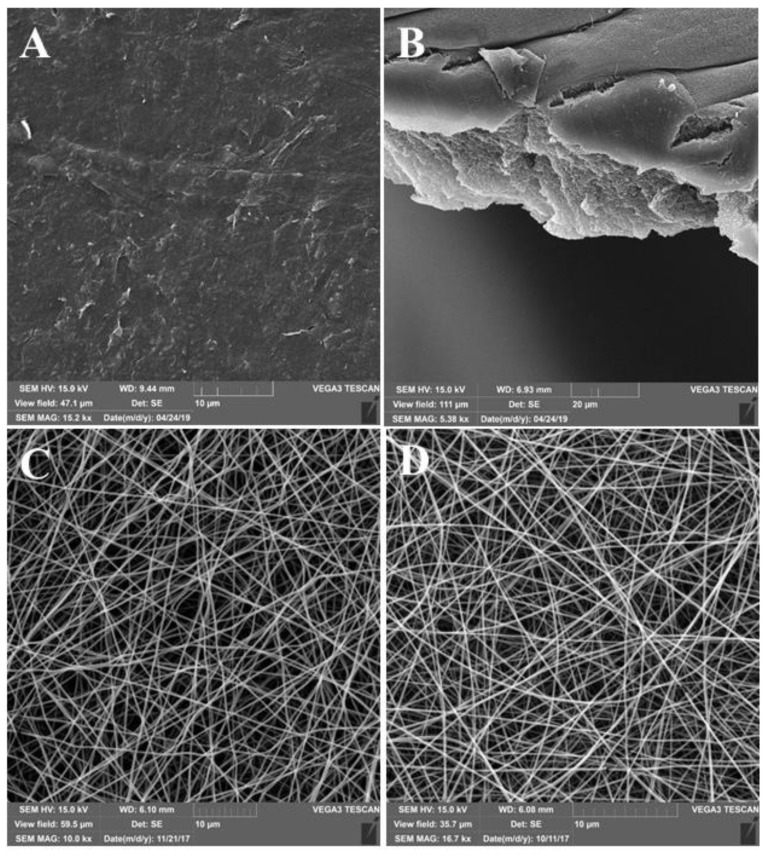
SEM-surface images of constructions: (**A**) SF-F; (**B**) section of SF-F; (**C**) SF-MS; and (**D**) SFG-MS.

**Figure 2:**
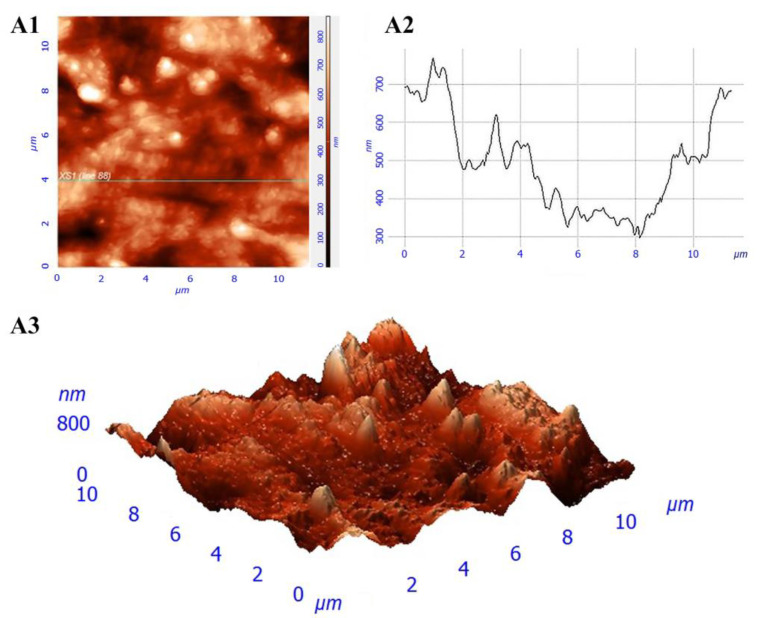
Film structure: (**A1**) surface image; (**A2**) surface profile along the specified line; and (**A3**) three-dimensional image of the surface.

The surfaces of the films had a micro- and nano-relief (Figure 1A,B). There were no pores in the revealed structure of the films. The roughness of the obtained films was 36.5 ± 10.6 nm (Figure 2).

Electrospun scaffolds were characterized by a microfibrous structure (Figure 1C,D). Fibers were randomly arranged in several layers that form a porous mat. The approximate thickness of the fiber was in the range of 300–700 nm.

**Figure 3:**
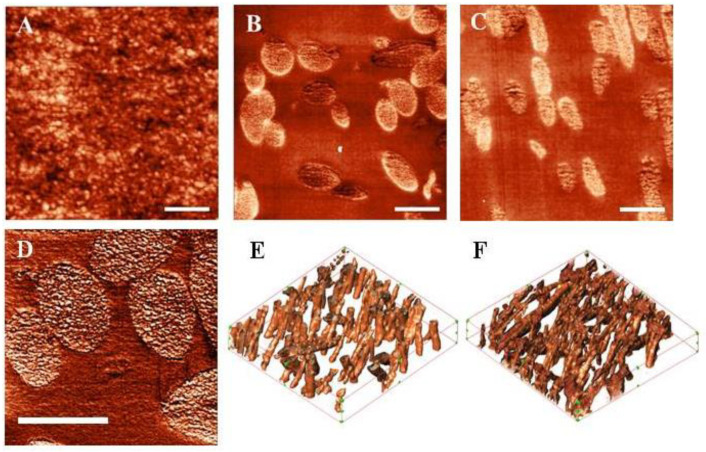
Internal structure of the constructions: (**A**) section surface of SF-F; (**B**) section surface of SF-MS; (**C**) section surface of SFG-MS; (**D**) single fiber section of SF-MS; (**E**) 3D reconstruction of SF-MS; and (**F**) 3D-reconstruction of SFG-MS. The scale bar on (**A**–**D**) is 1 μm. The scanning area on (**E**,**F**) images is 13.7 × 12.7 × 2.25 μm^3^, the number of sections is 15, and the thickness of the section is 150 nm.

The analysis of the internal structure of the constructions was performed using SPNT. The observed volume structures can be characterized as consisting of densely packed globule structures 10–30 nm in size (Figure 3A).

Surface images of sections of microfibrous scaffolds were obtained (Figure 3B,C). The obtained three-dimensional reconstructions (Figure 3E,F) of microfibrous scaffolds were used to calculate the volume porosity and the SA:V of the scaffold (Table 2). Pore dimensions may be estimated in the range 3–5 µm. There were no statistically significant differences between the structural parameters of the scaffolds.

Single polymer fibers of the scaffolds were characterized by an inhomogeneous internal structure (Figure 3D) and consisted of globules with sizes from 10 to 30 nm. This is consistent with the research on the internal structure of the films. It can be assumed that, in both cases, silk fibroin globules in the structure were detected.

### 3.2. Experiments with Cells

**Figure 4:**
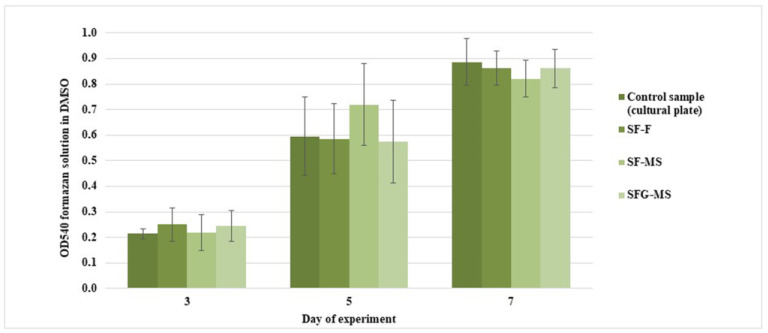
MTT assay on days 3, 5, and 7. The standard deviation values for 5 measurements are indicated.

The MTT assay has shown that the proliferative activity of cells did not decrease after the addition of construction samples in the incubation medium (Figure 4). This indicates the absence of a negative effect of constructions on the viability of cells. Thus, the constructions are suitable for further experiments.

**Figure 5:**
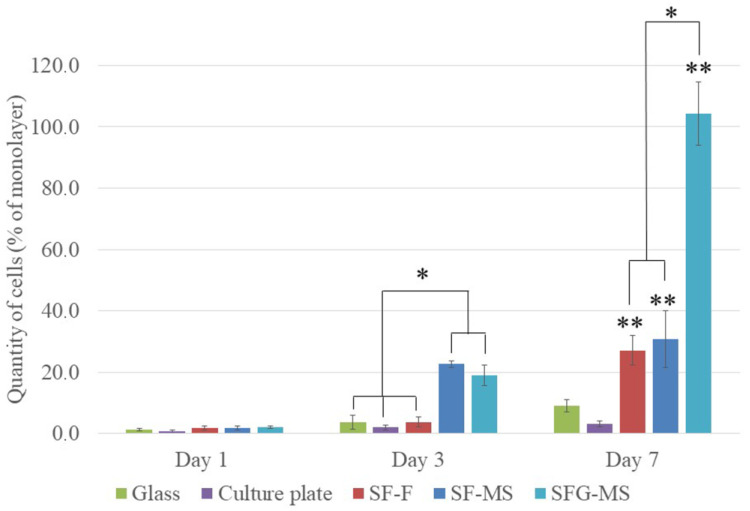
Adhesion (1 day) and proliferative activity (3 and 7 days) of mice 3T3 fibroblasts. The standard deviation values for 5 measurements are indicated. * indicates statistically significant differences between the experimental samples of constructions; ** marks statistically significant differences between the samples of constructions and the control samples.

Cell adhesion occurred in all samples with no differences in the cell adhesion process (Figure 5). The morphology of the cells was similar, and the cells formed filopodia and adhered tightly to the substrate, while the adhesion of the cells was uneven. There were no significant differences between the levels of cell adhesion in different samples.

The analysis of the proliferative activity of cells on the third day revealed increases in the levels of cell proliferative activity on microfibrous scaffolds (Figure 5, *). Cells penetrated into the deeper layers of the scaffolds during proliferation.

On the seventh day, the proliferative activity of the cell on constructions was significantly higher than on the control samples (Figure 5, **). The highest level of cell proliferative activity was observed on SFG-MS, in which cells formed a dense monolayer (Figure 5, *). The cell proliferative activity on SF-F and SF-MS did not differ significantly.

### 3.3. The Investigation of the Healing Process of the Full-Thickness Skin Wound of Wistar Rats

**Figure 6:**
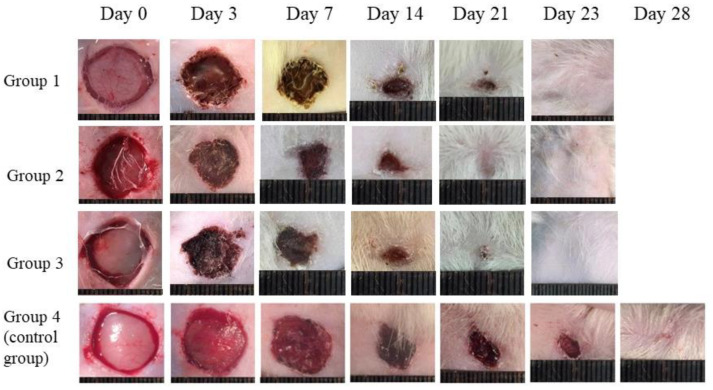
Images of wounds on the 0th, 3rd, 7th, 14th, 21st, 23rd and 28th days. Group 1: SF-F, group 2: SF-MS, and group 3: SFG-MS.

On the 0th, 3rd, 7th, 14th, 21st, 23rd, 28th and 40th days of the experiment, images of wounds were obtained (Figure 6). The images on the 40th day are not shown, due to the wound being healed in all the groups by this time, with the exception of the control group.

**Figure 7:**
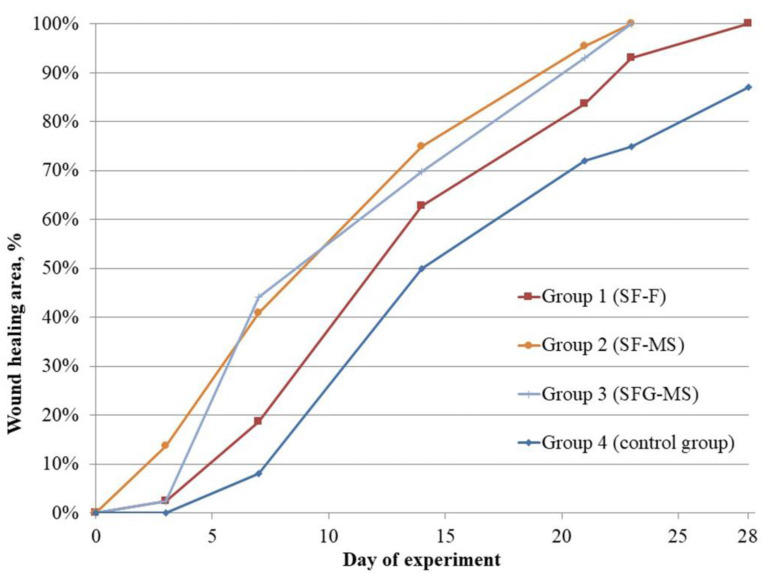
Healing curves of the full-thickness skin wounds.

To quantify the wound healing process, wound diameters were measured on the 0th, 3rd, 7th, 14th, 21st, 23rd, 28th and 40th days of the experiment (Table 3), and healing curves were plotted (Figure 7).

Constructions accelerated the healing process by an average of 17 days, as compared with the control group. No statistically significant differences were found between films and scaffolds in the healing rate.

**Figure 8:**
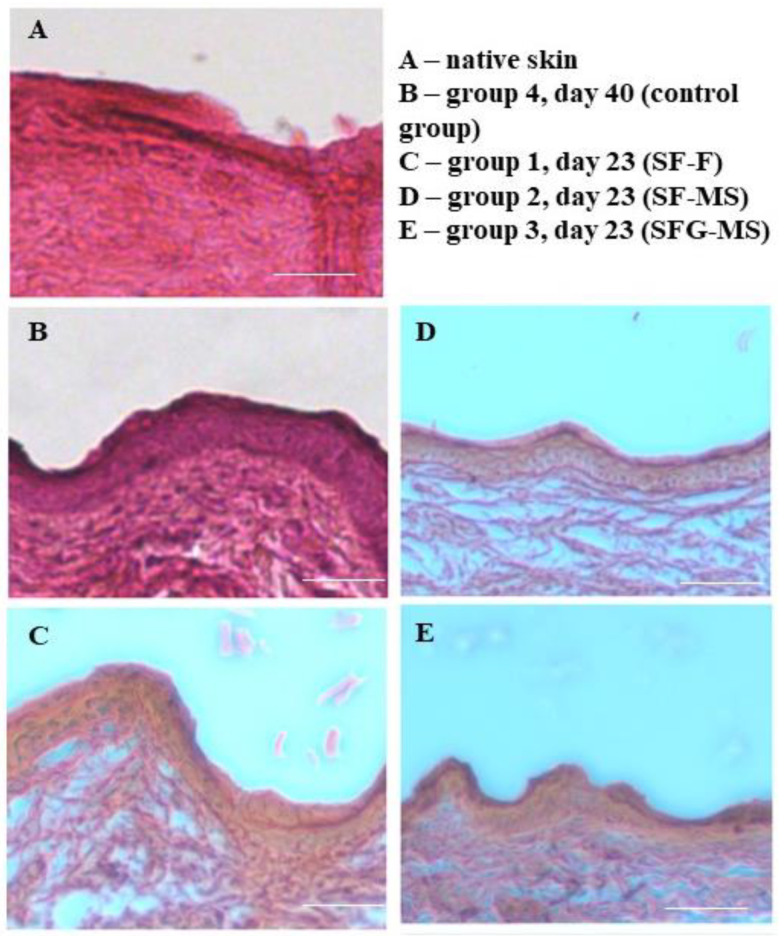
Histological evaluation in the area of complete wound healing (A = 100%), magnification ×100, and scale bar: 100 μm. (**A**) native skin; (**B**) group 4, day 40 (control group); (**C**) group 1, day 23 (SF-F); (**D**) group 2, day 23 (SF-MS); (**E**) group 3, day 23 (SFG-MS).

Qualitative analysis of the healing process was performed histologically. Samples of the skin were taken on the day of complete restoration of the skin in the wound area (A = 100%). In all analyzed sections, the presence of three layers of skin was revealed: the epidermis, dermis and hypodermis (Figure 8), which indicated successful wound healing. The morphology of the sections of the skin samples from experimental groups did not differ from the morphology of the sections of native rat skin. The epidermis mainly consists of keratinocytes, which form tight contacts. Rat skin samples from experimental groups 1–3 were characterized by the intensive proliferation of fibroblasts in the dermis, which indicates the restoration of this layer. There were no areas of inflammation in sections and no fragments of constructions, which is also an important indicator characterizing the successful healing process of the skin.

## 4. Discussion

Two types of constructions were developed for this research: films, which were obtained using the casting method, and microfibrous scaffolds, which were obtained using electrospinning method. Films were transparent and had thicknesses of 20–40 μm; microfibrous scaffolds were characterized by a milky-white color and a thickness of 10–30 μm.

Previously, the authors of this article conducted comparative studies of films, which were fabricated from polymer solutions with various solvents. The authors showed that films based on an aqueous solution do not have a toxic effect on cells, maintain cell adhesion and proliferation at a high level, and have a high regenerative potential [26,29].

Two groups of microfibrous scaffolds based on B. mori silkworm fibroin were obtained: constructions that contained only silk fibroin, and that contained gelatin as a composite additive. Gelatin is widely used in various research areas as a product of collagen denaturation [31,32,33,34,35]. However, a number of researchers have noted that the use of collagen can adversely affect the mechanical properties of constructions and that collagen dissolution can be associated with a number of technical difficulties (the limited solubility of collagen, the need to expose the polymer to active chemicals, etc.) [36,37]. Denaturation of collagen with subsequent hydrolysis leads to the formation of gelatin with different amino acid sequences depending on denaturation conditions [38]. In this study, type A gelatin with a positive charge at neutral pH was used to increase biocompatibility [39].

HFIP was selected as a solvent due to its high volatility for quick drying of the fibers. It is a key property for obtaining the scaffold with the correct structure since solvent pervasion on the cathode can cause the dissolution and disruption of the fibrous structure [31].

The determinant parameters for obtaining scaffolds using the electrospinning method are the concentration of the solution and its feed rate, the diameter of the needle, the distance between the needle and the cathode, and the voltage and its alteration allows us to standardize the structure of all microfibrous scaffolds.

In accordance with the main goal of this research, the properties of constructions, which provide the possibility of their utilization in regenerative medicine, were investigated.

The structure analysis of all the constructions was investigated using SEM and SPNT [40]. SEM revealed micro- and nano-reliefs in the form of roughnesses on the surface of the films [27]. The surface structure of the construction influences cell adhesion [25] since the presence of roughness on the substrate surface increases the surface area, which is available for cell adhesion. At the same time, there is an optimal level of the substrate roughness for each cell culture [41].

The internal structure of films was characterized using the novel method of SPNT, which has a higher resolution than SEM. This was uniform and consisted of tightly packed protein globules, which can be formed as a result of protein aggregation during the drying of the polymer solution. The analysis did not reveal nano- or micro-pores in the structure, which can have a significant effect on the regenerative potential of the film.

The non-porous film can maintain the necessary concentration of nutrients in the regeneration area and provide a barrier between injured tissues and surrounding tissues. The accumulation of nutrients in the damaged tissue area can stimulate the migration of healthy tissue cells and direct their proliferation, which accelerates regeneration [42]. This expands the prospects of using silk fibroin-based films as a drug delivery system, as well as for the regulation of the releasing rate of the active substances.

SEM allowed us to confirm the fibrous structures of scaffolds and to measure the thickness of the fibers, which was in the range of 300–700 nm. However, this method does not allow us to obtain detailed information about the structure and only allows us to compare the structure of scaffolds at the qualitative level. The unique novel data on the volume porosity of the scaffolds and the SA:V of such constructions were obtained using SPNT, which are important parameters for describing the structure of scaffolds [4]. These parameters, in various scaffolds, do not have statistically significant differences, which allows us to consider the structure of the scaffolds as similar and to exclude its effect on the differences of biological properties.

The porosity of the scaffolds affects not only the migration of cells in the structure, but also determines the swelling property of the scaffolds for macromolecules, which are necessary for the synthesis of extracellular matrix components using cells. In addition, the porosity of the scaffolds ensures the removal of toxic metabolic products, or their redistribution, which can ensure the restoration of extensive tissue damage [43,44,45].

The high resolution of SPNT ensures the analysis of the individual fiber structure [28]. SPNT revealed the heterogeneous internal structure of fibers, which can have a significant effect on cell proliferation in the structure of scaffolds and is similar to the internal structure of the films due to the similarity of polymer drying processes.

Further studies were aimed at investigating the constructions’ biocompatibility. None of the sample groups had a toxic effect on cells, which determined the suitability of the constructions’ samples for further research.

Adhesion and proliferation were evaluated on mice 3T3 fibroblasts, which have integrin receptors of various types, namely the ligand of RGD-sequences, which are present in gelatin. It was found that all of the obtained constructions are biocompatible, while the proliferative activity of the cells in the constructions increased during the experiment and its level was comparable to or higher than the level of the proliferative activity of cells in the control samples. This result can be explained by the optimal level of film roughness and biocompatible properties of silk fibroin [46].

The proliferative activity of cells on the 3rd day of the experiment in microfibrous scaffolds was significantly higher than in films. Various groups of researchers have shown that cell proliferation in the composition of fibrous structures is faster, since such a structure provides cell migration and creates a microenvironment close to the fibrous structure of the native extracellular matrix of the tissues [47]. The absence of the effect of increased cell adhesion and proliferative activity on microfibrous scaffolds containing gelatin in the composition can be associated with the inaccessibility of RGD-sequences in the structure of gelatin molecules from cell receptors due to silk fibroin [27]. On the 7th day of the experiment, the highest proliferative activity of the cells was observed on microfibrous scaffolds containing gelatin. Areas with a dense monolayer of cells were formed on these samples. Such an increase in proliferative activity can be associated with the stage degradation of silk fibroin and the emergence of the RGD-sequence. This corresponds to the fact that silk fibroin is widely researched as a depot for the controllable release of bioactive molecules, such as growth factors (EGF, GDNF), because of the stage degradation process [48,49].

The investigated properties of films and microfibrous scaffolds allowed us to consider them as promising wound dressings [30,50,51].

Differences in properties of constructions determined the course of the implantation process. Films were hygroscopic and were fixed easily on the wound surface without suturing; films were also able to retain moisture and did not require constant moisture during the implantation process. These properties facilitated the process of implantation, prevented the destruction of the film during implantation, and decreased the infliction and additional damages.

Microfibrous scaffolds have high permeability and hygroscopicity due to their porosity, which facilitated implantation, allowed us not to sew the scaffold and significantly reduced the operation time. Several scaffolds can be positioned at once in the wound area in order to create a multilayer structure. Additionally, the hemostatic effect of microfibrous scaffolds with gelatin was noted.

The analysis of the wound healing process dynamics revealed an acceleration caused by the microfibrous scaffolds, as compared to films from days 3 to 21 of the experiment. This can be associated with a microfibrous structure, which causes gradual wound healing. In the first stages of the healing process, the microfibrous structure allowed cells to migrate to the injured area more efficiently and to proliferate into the volume of the construction. The analysis of the results in the subsequent days of the experiment did not reveal differences in the healing rate between the constructions of similar compositions, which were obtained using different methods. The adhesion and proliferation of fibroblasts occurred faster and corresponded to the increase in roughness of the substrate [52]. This may be a possible reason for the more expressed effect of accelerated wound healing by films. Currently, methods of surface structure control are being developed to improve the biocompatibility for keratinocytes and fibroblasts [53]. The acceleration of the healing rate using films at a later healing stage can be attributed to the absence of porosity, which promotes the accumulation of bioactive substances in the area of injury. For example, the addition of gold nanoparticles into the volume of electrospun silk fibroin scaffold did not have a notable effect on skin healing [54] thus, it can be considered to have electrospinning method advantages.

The addition of gelatin into the composition of microfibrous scaffolds did not have the acceleration effect on the wound healing, but a feature of the wound healing dynamics was revealed, which is expressed by a prompt increase in the wound-healing rate at 3–7 days. Fibroblasts began to synthesize components of the extracellular matrix of the skin, including collagen, from the 3rd to the 15th day of the wound healing process [42]. The biodegradation of gelatin, as part of the construction, provides amino acids for cell metabolism, including collagen synthesis, which can stimulate the healing process.

Histological evaluation confirmed the high regenerative potential of the obtained constructions. In the wound areas, there was no inflammation or construction fragments, and there was a successful restoration of all layers of the skin. These results corresponded to the data that was obtained previously by other groups. It was shown that silk fibroin constructions were safe under acute dermal toxicity, acute dermal irritation and skin sensitization [55]. Additionally, extensive in vivo investigations and detailed histological evaluations of the silk fibroin scaffolds effect on the healing process showed suppression of inflammation, stimulation of re-epithelialization and reduction of the wound healing period and scar formation [56].

Thus, the obtained constructions can be considered as possible promising constructions for various tasks of tissue engineering and regenerative medicine.

## 5. Conclusions

In the course of the research, constructions with various micro- and nano-structures were obtained: films based on silk fibroin and microfibrous scaffolds based on silk fibroin and gelatin. The methods were developed to standardize the structural parameters of the constructions. The surface of the obtained films has a relief in the form of micro- and nano-roughness, and their internal structure is characterized by the presence of silk fibroin globules and the absence of pores. Microfibrous scaffolds contain randomly distributed fibers, and are characterized by high-volume porosity and SA:V. The internal structure of individual fibers in microfibrous scaffolds is similar to the internal structure of the films. Constructions are non-cytotoxic and maintain cell adhesion and proliferation at a high level. Films and microfibrous scaffolds are suitable for surgical manipulations in the model of the full-thickness skin wound healing. In this in vivo experimental model, constructions accelerated the healing of the skin by an average of 15 days (1.6 times), as compared to the control group; the morphology of the newly formed skin did not differ from the morphology of the native skin, which indicates a high regenerative potential of the constructions. Thus, films and microfibrous scaffolds are promising for further utilization in tissue engineering and regenerative medicine.

## Figures and Tables

**Table 1:** Description of the experimental groups. The number of animals in each group was 5.

Group Number	Wound Dressing Description
1	Silk fibroin aqueous solution with the protein concentration of 20 mg/mL, casting method (SF-F)
2	Silk fibroin HFIP-solution with the protein concentration of 50 mg/mL, electrospinning method (SF-MS)
3	Silk fibroin and gelatin HFIP-solution with the total protein concentration of 50 mg/mL which contains 70% of fibroin by total protein weight and 30% of gelatin by total protein weight, electrospinning method (SFG-MS)
4	(control)

**Table 2:** The structural parameters of microfibrous scaffolds. Values of the standard deviations for 6 independent measurements are presented.

Scaffold Description (Group Number)	Volume Porosity %	SA:V, μm^−1^
SF-MS (group 2)	81.3 ± 12.6	37.2 ± 9.7
SFG-MS (group 3)	86.4 ± 10.5	33.8 ± 7.4

**Table 3:** Dynamics of the healing process. The standard deviation values for 5 measurements are indicated. * indicates statistically significant differences between experimental and control groups.

Group Number	0 Day	3 Day	7 Day	14 Day	21 Day	23 Day	28 Day	40 Day
1	0	2 ± 1	19 ± 0.6 *	61 ± 1.5 *	84 ± 1.5 *	98 ± 1 *	100 *	
2	0	14 ± 1.5 *	41 ± 1.2 *	75 ± 0.6 *	95 ± 0.6 *	100 *		
3	0	2 ± 1.7	44 ± 1 *	70 ± 1.5 *	93 ± 0.6 *	100 *		
4	0	0	12.5 ± 1	50 ± 1.5	68 ± 1.5	75 ± 1.5	87.5 ± 1.7	100

## Data Availability

The data presented in this study are available on request from the corresponding author.
